# Network analysis of the associations between personality traits, cognitive functioning, and inflammatory markers in elderly individuals without dementia

**DOI:** 10.3389/fnagi.2023.1093323

**Published:** 2023-04-17

**Authors:** Thomas Bastelica, Louis-Ferdinand Lespine, Isabelle Rouch, Myriam Tadri, Jean-Michel Dorey, Marie-Pierre F. Strippoli, Thierry d'Amato, Armin von Gunten, Martin Preisig, Romain Rey

**Affiliations:** ^1^Centre Hospitalier Le Vinatier, Bron, France; ^2^INSERM, U1028; CNRS, UMR5292, Lyon Neuroscience Research Center, Psychiatric Disorders: from Resistance to Response Team, Lyon, France; ^3^INSERM U1219, Bordeaux Population Health Centre Recherche (BPH), Bordeaux, France; ^4^Memory Clinical and Research Center of Saint Etienne (CMRR), Neurology Unit, University Hospital of Saint Etienne, Saint-Etienne, France; ^5^Lausanne University Hospital and University of Lausanne, Lausanne, Switzerland; ^6^INSERM, Brain Dynamics and Cognition, Lyon Neuroscience Research Center, Lyon, France

**Keywords:** cognition, personality, inflammation, elderly, network analysis

## Abstract

**Introduction:**

Lower cognitive functioning in old age has been associated with personality traits or systemic inflammatory markers. Associations have also been found between personality traits and inflammatory markers. However, no study has explored the inter-relationships between these three components simultaneously. The present study aims to better understand the inter-relationships among personality traits, inflammatory markers, and cognitive performance in elderly individuals without dementia.

**Methods:**

This study utilizes a network analysis approach, a statistical method that allows visualization of the data’s unique pairwise associations. We performed a cross-sectional analysis on 720 elderly individuals without dementia, using data from Colaus|PsyColaus, a population-based study conducted in Lausanne, Switzerland. The Revised NEO Five-Factor Inventory (NEO-FFI-R) was used to assess personality traits, and interleukin (IL)-1β, IL-6, tumor necrosis factor-α (TNF-α), and C-reactive protein (CRP) were used as peripheral inflammatory markers. Cognitive domains were investigated using the Mini-Mental State Examination (MMSE), the Verbal Fluency Test, the Stroop Test, the DO40, and the Free and Cued Selective Reminding (FCSR) test.

**Results:**

Openness was associated with verbal fluency and Agreeableness with immediate free recall. In contrast, no association between inflammatory markers and personality traits or cognition was identified.

**Discussion:**

In elderly individuals without dementia, a high level of Openness or Agreeableness was associated with executive functioning/semantic memory and episodic memory, respectively.

## 1. Introduction

Cognitive aging is characterized by a decline in cognitive functions, such as decreased memory, problem-solving competence, executive abilities, or processing speed (Harada et al., 2013). Cognitive functioning varies significantly across individuals and is associated with elderly adults’ functional status and independence, presenting challenges to the healthcare system (Moritz et al., 1995; Njegovan et al., 2001). Several biological, psychosocial, and environmental factors have been linked to cognitive functioning in healthy elderly people (Hendrie et al., 2006), including personality traits and inflammation (Sartori et al., 2012; Curtis et al., 2015).

Recently, many studies have investigated the relationship between personality traits and cognitive performance (Curtis et al., 2015). Personality dimensions are often assessed according to the five-factor model (McCrae and Costa, 1987), including Neuroticism, Extraversion, Openness to experience, Agreeableness, and Conscientiousness. Numerous cross-sectional studies carried out in healthy middle-aged and elderly people revealed that individuals with higher levels of Openness tend to perform better in several cognitive domains, such as general cognitive ability (Austin et al., 2002; Booth et al., 2006; Simon et al., 2020), reasoning (Soubelet and Salthouse, 2011; Simon et al., 2020), and episodic memory (Booth et al., 2006; Soubelet and Salthouse, 2011; Aiken-Morgan et al., 2012). Similarly, a correlation between higher Conscientiousness and better cognitive performance has been found (Soubelet and Salthouse, 2011; Graham and Lachman, 2014; Simon et al., 2020). In contrast, Extraversion has been negatively associated with several cognitive abilities (Austin et al., 2002; Baker and Bichsel, 2006; Soubelet and Salthouse, 2011; Graham and Lachman, 2014; Simon et al., 2020). Several studies revealed a negative association between Neuroticism and general cognitive functioning (Jorm et al., 1993; Austin et al., 2002; Boyle et al., 2010), executive functions (Booth et al., 2006; Williams et al., 2010), and episodic memory (Jorm et al., 1993; Meier et al., 2002; Aiken-Morgan et al., 2012), while others did not (Jelicic et al., 2002; Baker and Bichsel, 2006). Finally, mixed results have been reported regarding Agreeableness (Curtis et al., 2015). Some authors observed positive associations with memory/attentional performance (Aiken-Morgan et al., 2012), whereas others found no association (Austin et al., 2002; Baker and Bichsel, 2006; Soubelet and Salthouse, 2011; Chapman et al., 2012; Curtis et al., 2015) or a negative association with reasoning and executive functions (Baker and Bichsel, 2006; Soubelet and Salthouse, 2011; Graham and Lachman, 2014; Ouanes et al., 2017).

Studies have shown inconsistent results concerning associations between systemic inflammatory markers and the evolution of cognitive functions. For example, some cross-sectional and longitudinal studies involving people without dementia found that higher levels of interleukin (IL)-6 and C-reactive protein (CRP) are associated with an increased risk for all causes of dementia (Darweesh et al., 2018) and cognitive decline. More specifically, several studies showed elevated levels of IL-6 and CRP to be associated with global cognitive decline (Weaver et al., 2002; Yaffe et al., 2003; Jordanova et al., 2007) and lower performance in executive functions (Schram et al., 2007; Trollor et al., 2012) and memory (Elwan et al., 2003; Teunissen et al., 2003; Komulainen et al., 2007; Schram et al., 2007). However, other studies failed to replicate these findings (Dik et al., 2005; Alley et al., 2008; Baune et al., 2008; Todd, 2017; Keegan et al., 2018; Fard et al., 2020).

Finally, numerous studies, primarily focusing on middle-aged adults (mean age ranging from 45.6 to 67.9 years), have assessed associations between personality traits and systemic inflammatory markers (Armon et al., 2013; Turiano et al., 2013; Allen and Laborde, 2017; Graham et al., 2018). Studies have also found that high levels of Openness and Conscientiousness are linked to lower levels of IL-6 and CRP (Armon et al., 2013; Turiano et al., 2013; Graham et al., 2018; Wright et al., 2022), whereas Neuroticism has a positive correlation with these inflammatory markers (Sutin et al., 2010; Graham et al., 2018; Wright et al., 2022). Few studies have addressed this question in healthy people over 65 years, and results have been inconsistent (Chapman et al., 2011; Mõttus et al., 2013).

The present study aims to better understand the inter-relationships between personality traits, systemic inflammatory markers, and cognitive performances using a network analysis approach. This approach has been specifically developed to explore complex interactions and has recently received increased attention in psychiatric research (Fried and Cramer, 2017; Contreras et al., 2019). Network analysis provides indications to causal structure through conditional independencies and can be used to connect different scientific disciplines to each other (Borsboom et al., 2021). Thus, a network analysis was used to identify the unique pairwise associations between cognition function, personality traits, and inflammation while all other variables in the network were controlled. A better understanding of these relationships is critical to improving prevention and treatment and unraveling the mechanisms involved in cognitive functioning during aging. Since previous results on this research topic are inconsistent in people over 65, we had no strong assumptions. Thus, this study is exploratory.

## 2. Materials and methods

### 2.1. Study design and participants

A cross-sectional analysis was conducted using data from the first follow-up of CoLaus|PsyCoLaus, a population-based cohort designed to study mental disorders and cardiovascular risk factors as well as their associations in the community (see Firmann et al., 2008; Preisig et al., 2009). The CoLaus|PsyCoLaus study took place between 2003 and 2006 and included a random sample of 6,734 participants (age range: 35–75 years) selected from the residents of Lausanne, Switzerland. The baseline somatic assessment for the CoLaus study was conducted between June 2003 and May 2006 (Firmann et al., 2008). One year later, all participants aged 35–66 were invited to participate in the PsyCoLaus, the psychiatric evaluation (Preisig et al., 2009). Afterward, between 2010 and 2013, 4,004 participants out of all invited participants took part in the first follow-up, consisting of physical and psychiatric evaluations.

### 2.2. Inclusion/exclusion criteria

**Figure 1:**
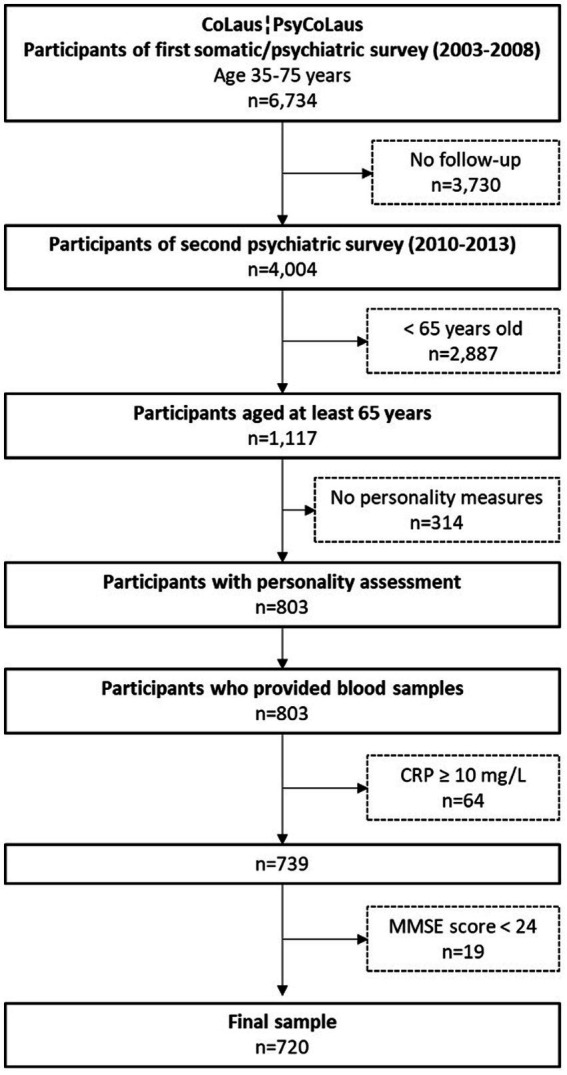
Flow chart of included participants.

The selection criteria of the sample used for the present study are presented in Figure 1. The included participants (1) underwent a neurocognitive assessment (participants aged 65 or older), (2) completed the personality questionnaire, and (3) provided blood samples. All data used in this study were collected during the first follow-up, resulting in 803 participants. Among them, we excluded 64 participants with CRP levels >10 mg/l, indicating a high likelihood of acute inflammation (Myers et al., 2004; Bell et al., 2017). Finally, individuals with an MMSE score < 24, suggesting dementia, were excluded, which resulted in a final sample size of 720 participants.

### 2.3. Ethics statement

The local Ethics Commission approved the CoLaus|PsyCoLaus study (www.cer-vd.ch; project number PB_2018–00038, reference 239/09). The approval was renewed for the first (reference 33/09) and second (reference 26/14) follow-ups. The study was performed in agreement with the Helsinki Declaration and its former amendments in accordance with applicable Swiss legislation. All participants signed written informed consent.

### 2.4. Measures

#### 2.4.1. Cognition

Cognitive functioning was assessed by trained master-level psychologists. The assessment included the following instruments:

- *Global cognitive performance*. Global cognitive performance was assessed using the Mini-Mental State Examination (MMSE) (Folstein et al., 1975), the most commonly used screening tool for global cognitive impairment. MMSE scores range from 0 to 30, with a higher score indicating superior performance.
- *Memory*. Memory was assessed using the French adaptation of the Free and Cued Selective Reminding (FCSR) test (Van der Linden et al., 2004). The FCSR test includes a learning list of 16 written words presented with a semantic cue to control for memory encoding. First, participants were asked to retrieve the words spontaneously and then with the help of the semantic cue. Three trials were conducted. Finally, their free and cued delayed recall ability was examined after 30 min. Total immediate free recall scores were used in this study (the sum of three trials). Scores ranged from 0 to 48, with a higher score indicating better performance.
- *Generativity/semantic memory*. *Generativity (executive functions) and semantic memory* were evaluated using the Verbal Fluency Test and the letter (phonemic) and category (semantic) fluency tasks (Cardebat et al., 1990). A higher total score (the sum of the phonemic and semantic scores) indicated more advanced performance.
- *Inhibition*. Executive functions, including inhibition, were assessed using the Stroop Test (Stroop, 1935). The number of correct items during the interference condition range from 0 to 24, with a higher score indicating better performance.
- *Language abilities.* Language abilities were assessed using the DO40 picture-naming test (Deloche and Hannequin, 1997). DO40 scores range from 0 to 40, with a higher score indicating superior performance.

#### 2.4.2. Personality

Personality traits were measured using the French version of the Revised NEO Five-Factor Inventory (NEO-FFI-R), which has been validated in Switzerland (Aluja et al., 2005). The NEO-FFI-R is a short version of the Revised NEO Personality Inventory, measuring the five factor-analytically derived personality dimensions: Neuroticism, Extraversion, Openness, Agreeableness, and Conscientiousness. Participants respond to 60 questions using a 5-point Likert scale, ranging from 1 (strongly disagree) to 5 (strongly agree). For each of the five dimensions, a score is computed by summing the scores that correspond to each category (Costa and McCrae, 1992).

#### 2.4.3. Inflammation biomarkers

Serum blood samples were used to assess systemic levels of high-sensitive CRP, IL-6, IL-1β, and tumor necrosis factor-α (TNF-α). These markers were selected because they are implicated in key biological pathways, especially in cardiometabolic conditions (Ridker et al., 2000, 2017; Pradhan et al., 2002; Danesh et al., 2008). Moreover, they are promising candidates as peripheral inflammatory biomarkers of cognition in older adults without dementia (Li and Yu, 2017). Blood samples were collected as part of the physical investigation, occurring approximately 1 year before the psychiatric evaluation: median length of 12 months (Interquartile range: 4.5 months). See Supplementary material for details.

#### 2.4.4. Covariates

Based on previous research (Duivis et al., 2013; Haapakoski et al., 2015; Wagner et al., 2019), covariates included age; self-reported sex at birth (female = 0, male = 1); education (primary = 0, secondary and higher = 1); depressive symptoms, assessed by the French version of the Center for Epidemiologic Studies Depression Scale (CES-D) (Morin et al., 2011); alcohol intake, a self-reported measure (in units) of weekly consumption; current smoking, self-reported status measure (no = 0, yes = 1); physical activity was described as occurring at least 20 min >2/week (no = 0, yes = 1); and waist-to-hip ratio (WHR). Due to age-related changes in height and body composition in older adults, WHR (a marker of visceral obesity) was considered more relevant regarding health-related risk than markers of generalized obesity, such as body mass index (Srikanthan et al., 2009).

### 2.5. Statistical analyses

#### 2.5.1. Data preparation

Missing data, varying from 0.4 to 13.1%, were imputed using multivariate imputations by chained equations (MICE) (van Buuren and Groothuis-Oudshoorn, 2011). A total of 10 separate imputation data sets were created. The final data set was based on the median (for continuous variables) or the mode (for categorical variables) of the 10 imputed data sets (see Supplementary Table 1). The FCSR test scores for free recall 1, free recall 2, and free recall 3 were summed. Additionally, the phonemic verbal fluency and semantic verbal fluency scores were added. WHR was calculated by dividing waist circumference by hip circumference. We used the statistical software R (version 3.4.4) to carry out the statistical analyses. Skewed distributions of IL-6, IL-1β, TNF-α, CRP, CES-D, and alcohol variables were normalized using the non-paranormal transformation (Liu et al., 2009). Because the Stroop interference index, DO40, and MMSE remained highly skewed even after the non-paranormal transformation, these variables were dichotomized based on their respective median (i.e., test values < median were coded as 0; test values = median were coded as 1, corresponding to the maximum score).

#### 2.5.2. Network estimation

Due to the exploratory nature of the present research, we specifically chose network analysis, which is considered an exploratory and hypothesis-generating method, providing a flexible framework for examining and visualizing the relationships among variables. In other words, network analysis can estimate complex patterns of relationships between variables. In network models, variables are considered “nodes” and “edges”; between nodes are conditional dependence relations that can be understood as partial correlations (i.e., estimates of the strength of relationships between variables after the effects of other variables in the network are controlled for). Given that our data consisted of both categorical and continuous variables, we estimated mixed graphical models (MGMs) with the R-package *mgm* (Haslbeck and Waldorp, 2020). To maximize specificity and avoid false positive findings, *mgm* uses the least absolute shrinkage and selection operator (LASSO, Tibshirani, 1996), leading to a sparse network structure, constituting standard practice in the network literature (Epskamp and Fried, 2018). The LASSO shrinks all edge weights and sets small weights to zero. The strength of the penalty is controlled by a parameter λ, which we selected using the extended Bayesian information criterion (EBIC; Foygel and Drton, 2010). The EBIC has a tuning parameter γ, which we set to zero to estimate more edges (i.e., the network has a higher sensitivity). It is important to note that the network will be sparser than a partial correlation network that does not employ any form of regularization; setting γ to 0 indicates that the EBIC reduces to the standard BIC, which still prefers simple models. This statistical methodology was inspired by Fried et al. (2020). Two networks were estimated. The first network (Network A) consisted of 14 nodes, including cognitive variables (n = 5), personality traits (n = 5), and inflammation markers (n = 4). The second network (Network B) included additional covariates (n = 8) for a total of 22 nodes.

We used the R-package *qgraph* to visualize the network structures, where green edges represent positive associations among variables and red edges depict negative associations. We used bootstrapping routines implemented in the package *bootnet* (Epskamp et al., 2018) to gain information on the precision of edge estimates (see Supplementary Tables 2, 3 and Supplementary Figures 1, 2).

**Figure 2:**
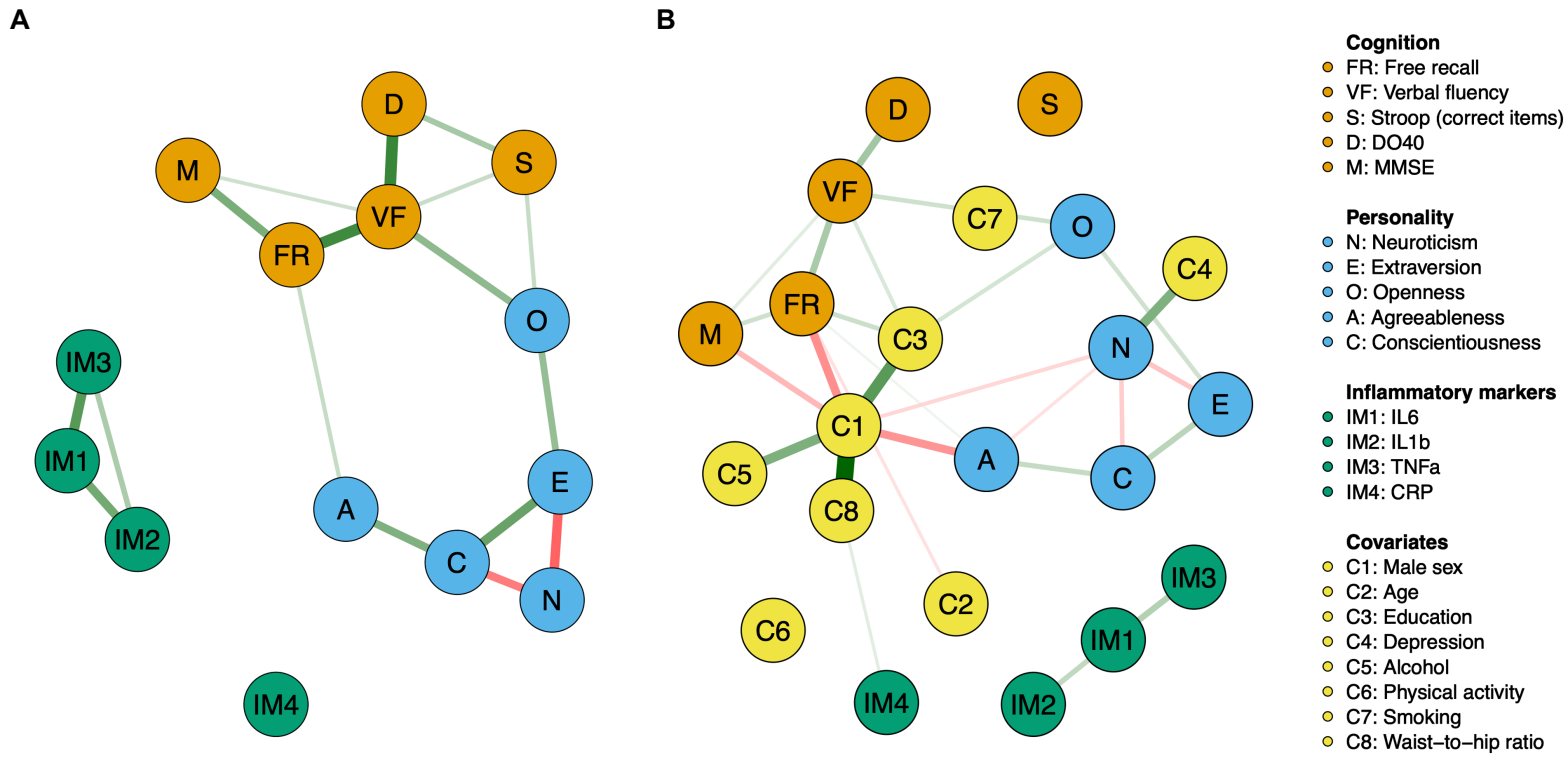
Network displaying the relationships between cognition, personality, and inflammatory markers before **(A)** and after **(B)** controlling for covariates. Green edges indicate positive partial correlations, while red edges indicate negative partial correlations. The thickness of the line indicates the strength of the relationship.

## 3. Results

### 3.1. Sample characteristics

The sample included 429 females (59.6%) and 291 males (40.4%). The mean age was 71.2 years (SD = 4.6; range = 65–85). Two hundred thirty-eight participants (33.1%) had a primary education level, and 482 participants (66.9%) had a secondary education level or higher. The characteristics of the sample are described in Table 1.

**Table 1:** Sample characteristics (*n* = 720).

Age, mean (SD) [min – max]	71.2 (4.6) [65–85]
Self-reported sex at birth	
Female, *n* (%)	429 (59.6)
Male, *n* (%)	291 (40.4)
Ethnicity	
Caucasian, *n* (%)	704 (97.8)
Non-Caucasian, *n* (%)	16 (2.2)
Education	
Primary, *n* (%)	238 (33.1)
Secondary and higher, *n* (%)	482 (66.9)
WHR, mean (SD) [min – max]	0.90 (0.10) [0.70–1.22]
CES-D, median (1st–3rd quartiles) [min – max]	8 (4–14) [0–51]
Alcohol intake (weekly units), median (1st–3rd quartiles) [min – max]	4 (1–8) [0–48]
Current smoking	
No, *n* (%)	620 (86.1)
Yes, *n* (%)	100 (13.9)
Physically active (at least 20 min >2/week)	
No, *n* (%)	531 (73.8)
Yes, *n* (%)	189 (26.3)
	
MMSE (global cognitive performance), median (1st-3rd quartiles)	30 (29–30) [24–30]
MMSE recoded	
< 30, *n* (%)	242 (33.6)
= 30, *n* (%)	478 (66.4)
FCSR Total immediate free recall (memory), mean (SD) [min – max]	30.3 (6.3) [7–45]
Verbal Fluency Test (Generativity/ semantic memory), mean (SD) [min – max]	51.0 (13.7) [19–109]
Stroop (inhibition), median (1st–3rd quartiles) [min – max]	24 (23–24) [7–24]
Stroop recoded	
< 24, *n* (%)	226 (31.4)
= 24, *n* (%)	494 (68.6)
DO40 (language abilities), median (1st–3rd quartiles) [min – max]	40 (40–40) [19–40]
DO40 recoded	
< 40, *n* (%)	93 (12.9)
= 40, *n* (%)	627 (87.1)
Neuroticism, mean (SD) [min – max]	17.5 (6.8) [0–39]
Extraversion, mean (SD) [min – max]	27.2 (6.0) [6–45]
Openness, mean (SD) [min – max]	29.0 (5.6) [11–47]
Agreeableness, mean (SD) [min – max]	33.7 (5.2) [18–48]
Conscientiousness, mean (SD) [min – max]	34.7 (5.4) [15–48]
IL-6, median pg./ml (1st–3rd quartiles) [min – max]	2.57 (1.07–8.06) [0.1–931]
IL-1β, median pg./ml (1st–3rd quartiles) [min – max]	0.43 (0.1–1.82) [0.1–158.9]
TNF-α, median pg./ml (1st–3rd quartiles) [min – max]	5.00 (2.96–8.51) [0.23–420]
CRP, median mg/l (1st–3rd quartiles) [min – max]	1.60 (0.80–3.00) [0.2–9.9]

WHR, Waist-to-hip ratio; CES-D, Center for Epidemiologic Studies Depression scale; MMSE, Mini-Mental State Examination; FCSR, Free and Cued Selective Reminding (FCSR) test; DO40, Picture-naming test; IL-6, Interleukin-6; IL-1β, Interleukin-1β; TNF-α, Tumor Necrosis Factor-α; CRP, C-reactive protein; SD, standard deviation.

### 3.2. Network output

#### 3.2.1. Partial correlations among inflammatory markers

In both networks, inflammatory markers IL-6, IL-1β, and TNF-α were related, with the strongest correlations between IL-6 and TNF-α (*r*s = 0.26 and 0.25 in Network A and Network B, respectively), and IL-6 and IL-1β (*r*s = 0.22 and 0.21 in Network A and Network B, respectively). TNF-α and IL-1β were also positively associated (*r*s = 0.13 and 0.11 in Network A and Network B, respectively). However, CRP had no relationship with any other markers. These network outputs are concordant with Spearman’s correlation coefficients computed on raw data (Table 2); however, we found evidence for CRP to be significantly but weakly associated with IL-6 (*r* = 0.11, *p* = 0.002) and TNF-α (*r* = 0.08, *p* = 0.042) (for a visualization of the Spearman’s correlations, see Supplementary Figure 3).

**Table 2:** Spearman’s correlation coefficients (*p*-value) between the inflammatory markers.

	IL-6	IL-1β	TNF-α	CRP
IL-6	—			
IL-1β	0.34 (< 0.001)	—		
TNF-α	0.40 (< 0.001)	0.25 (< 0.001)	—	
CRP	0.11 (0.002)	−0.07 (0.067)	0.08 (0.042)	—

IL-6, Interleukin-6; IL-1β, Interleukin-1β; TNF-α, Tumor Necrosis Factor-α; CRP, C-reactive protein.

**Table 3:** Partial correlations matrix of network A (cognition, personality, and inflammatory markers).

	FR	VF	S	D	M	N	E	O	A	C	IL-6	IL-1β	TNF-α	CRP
Free Recall (FR)														
Verbal Fluency (VF)	0.30													
Stroop (S)	0.09	0.07												
DO40 (D)	0	0.31	0.14											
MMSE (M)	0.21	0.08	0	0										
Neuroticism (N)	0	0	0	0	0									
Extraversion (E)	0	0	0	0	0	−0.24								
Openness (O)	0	0.18	0.09	0	0	0	0.17							
Agreeableness (A)	0.09	0	0	0	0	−0.08	0	0						
Conscientiousness (C)	0	0	0	0	0	−0.21	0.24	0	0.20					
IL-6 (IM1)	0	0	0	0	0	0	0	0	0	0				
IL-1β (IM2)	0	0	0	0	0	0	0	0	0	0	0.22			
TNF-α (IM3)	0	0	0	0	0	0	0	0	0	0	0.26	0.13		
CRP (IM4)	0	0	0	0	0	0	0	0	0	0	0	0	0	

DO40, Picture-naming test; MMSE, Mini-Mental State Examination; IL-6, Interleukin-6; IL-1β, Interleukin-1β; TNF-α, Tumor Necrosis Factor-α; CRP, C-reactive protein; 0, for likely spurious correlations LASSO regularization led to parameter estimates of exactly zero.

**Table 4:** Partial correlations matrix of network B (cognition, personality, inflammatory markers, and covariates).

	FR	VF	S	D	M	N	E	O	A	C	IL-6	IL-1β	TNF-α	CRP	C1	C2	C3	C4	C5	C6	C7	C8
Free Recall (FR)																						
Verbal Fluency (VF)	0.29																					
Stroop (S)	0	0																				
DO40 (D)	0	0.30	0																			
MMSE (M)	0.17	0.10	0	0																		
Neuroticism (N)	0	0	0	0	0																	
Extraversion (E)	0	0	0	0	0	−0.19																
Openness (O)	0	0.17	0	0	0	0	0.16															
Agreeableness (A)	0.05	0	0	0	0	−0.10	0	0														
Conscientiousness (C)	0	0	0	0	0	−0.17	0.23	0	0.20													
IL-6 (IM1)	0	0	0	0	0	0	0	0	0	0												
IL-1β (IM2)	0	0	0	0	0	0	0	0	0	0	0.21											
TNF-α (IM3)	0	0	0	0	0	0	0	0	0	0	0.25	0.11										
CRP (IM4)	0	0	0	0	0	0	0	0	0	0	0	0	0									
Male sex (C1)	−0.37	0	0	0	−0.23	−0.13	0	0	−0.35	0	0	0	0	0								
Age (C2)	−0.10	0	0	0	0	0	0	0	0	0	0	0	0	0	0							
Education (C3)	0.17	0.13	0	0	0	0	0	0.14	0	0	0	0	0	0	0.55	0						
CES-D (C4)	0	0	0	0	0	0.43	0	0	0	0	0	0	0	0	0	0	0					
Alcohol (C5)	0	0	0	0	0	0	0	0	0	0	0	0	0	0	0.43	0	0	0				
Physical activity (C6)	0	0	0	0	0	0	0	0	0	0	0	0	0	0	0	0	0	0	0			
Smoking (C7)	0	0	0	0	0	0	0	0	0	0	0	0	0	0	0	0	0	0	0	0		
Waist-to-hip ratio (C8)	0	0	0	0	0	0	0	0	0	0	0	0	0	0.09	0.85	0	0	0	0	0	0	

DO40, Picture-naming test; MMSE, Mini-Mental State Examination; IL-6, Interleukin-6; IL-1β, Interleukin-1β; TNF-α, Tumor Necrosis Factor-α; CRP, C-reactive protein; CES-D, Center for Epidemiologic Studies Depression scale; 0, for likely spurious correlations LASSO regularization led to parameter estimates of exactly zero.

#### 3.2.2. Partial correlations among personality traits

Neuroticism was negatively associated with Extraversion (*r*s = −0.24 and − 0.19 in Network A and Network B, respectively), Conscientiousness (*r*s = −0.21 and − 0.17 in Network A and Network B, respectively), and Agreeableness (*r*s = −0.08 and − 0.10 in Network A and Network B, respectively). Conscientiousness was positively associated with Extraversion (*r*s = 0.24 and 0.23 in Network A and Network B, respectively) and Agreeableness (*r*s = 0.20 in both networks). Extraversion and Openness were also positively associated (*r*s = 0.17 and 0.16 in Network A and Network B, respectively).

#### 3.2.3. Partial correlations among cognitive variables

Similarly, cognitive variables were interrelated with positive associations between verbal fluency and DO40 (*r*s = 0.31 and 0.30 in Network A and Network B, respectively), verbal fluency and free recall (*r*s = 0.30 and 0.29 in Network A and Network B, respectively), free recall and MMSE (*r*s = 0.21 and 0.17 in Network A and Network B, respectively), and verbal fluency and MMSE (*r*s = 0.08 and 0.10 in Network A and Network B, respectively). In Network A (i.e., without covariates), Stroop was positively associated with DO40 (*r* = 0.14), free recall (*r* = 0.09), and verbal fluency (*r* = 0.07). However, Stroop-related edges disappeared when corrected for covariates (Network B).

#### 3.2.4. Relationships between inflammation, cognition, and personality

Figure 2 shows the relationships between cognitive variables, personality traits, and inflammatory markers before (A) and after (B) controlling for covariates. Partial correlation matrices are reported in Tables 3, 4.

Cognition was connected to personality through Openness and Agreeableness. Openness was positively associated with verbal fluency (*r*s = 0.18 and 0.17 in Network A and Network B, respectively) and the Stroop index (*r* = 0.09), while Agreeableness was positively connected to free recall (*r*s = 0.09 and 0.05 in Network A and Network B, respectively). Although a positive association emerged between Openness and the Stroop index in Network A (*r* = 0.09), the link disappeared when corrected for covariates (Network B). Importantly, no relationship emerged between inflammatory markers and cognitive or personality variables.

Regarding associations with covariates (Network B), the male sex was associated with higher WHR (*r* = 0.85), education (*r* = 0.55), and alcohol intake (*r* = 0.43). The male sex was also associated with lower scores on free recall (*r* = −0.37), Agreeableness (*r* = −0.35), MMSE (*r* = −0.23), and Neuroticism (*r* = −0.13). Increasing age was associated with lower free recall (*r* = −0.10). Education level was positively associated with free recall (*r* = 0.17) and verbal fluency (*r* = 0.13). CES-D scores were only associated with Neuroticism (*r* = 0.43), and CRP was positively related to WHR (*r* = 0.09).

Stability analysis outputs are reported in Supplementary Tables 2, 3 and Supplementary Figures 1, 2. Some edges were estimated reliably (e.g., non-zero edges in all or nearly 1,000 bootstrapped samples), but there was considerable variability in the edge parameters across the bootstrapped models.

## 4. Discussion

To the best of our knowledge, this is the first study to assess the relationships between personality traits, inflammatory markers, and cognitive performance simultaneously. We found that the personality dimensions Openness and Agreeableness were positively associated with elevated scores on verbal fluency and episodic memory, respectively, whereas no association emerged between inflammatory markers and cognitive performance or personality traits.

### 4.1. Personality traits and cognition

Although previous studies on associations between personality traits and cognitive performance have provided inconsistent results, the observed association between Agreeableness, characterized by being trusting, sympathetic, and altruist (Costa and McCrae, 1992), and immediate free recall is consistent with the previously reported association between Agreeableness, learning, and memory in healthy elderly African Americans (Aiken-Morgan et al., 2012). This finding is also in conceptual agreement with a meta-analysis showing that higher Agreeableness could be a protective factor against Alzheimer’s disease (AD) (Terracciano et al., 2014). Notably, in the present study, the link between Agreeableness and memory decreased after introducing covariates in the network, especially the male sex, which was negatively associated with both Agreeableness and free recall.

A positive association was found between Openness and inhibition according to the Stroop test. However, this association disappeared after adjusting for covariates. Furthermore, Openness was positively associated with verbal fluency, suggesting that elderly individuals with increased Openness tend to have better generativity/semantic memory performances. Our findings are in accordance with previous results in healthy elderly people (Chapman et al., 2017; Crane et al., 2020) and in patients with prodromal and mild AD (Rouch et al., 2019). In contrast, other studies did not find any association between these variables (Booth et al., 2006; Boyle et al., 2010; Williams et al., 2010). A previous article based on a partially overlapping sample derived from the same cohort found no association between Openness and verbal fluency (Ouanes et al., 2017). Different definitions of what constitutes the verbal fluency domain, potentially leading to the measure of distinct constructs, may explain the discrepant results between the two studies. For instance, Ouanes et al. used a composite score relying on the fluency task and the DO40 picture-naming test, a denomination test evaluating linguistic abilities (Deloche and Hannequin, 1997). In contrast, we used the phonemic and semantic tasks, which involve executive function and semantic memory (Crawford and Henry, 2005). The link between Openness and cognition in old age may be explained, at least in part, by the cognitive reserve hypothesis (Curtis et al., 2015), which states that some favorable environmental factors, such as cognitive engagement (e.g., cognitive activities, social activities) could protect against age-related cognitive decline (Stern, 2003, 2012; Adam et al., 2013; Andel et al., 2015). Since individuals with high Openness are more likely to be engaged in stimulating activities across their lifespan (e.g., reading books or newspapers, working on crossword puzzles, etc.), they may have a greater cognitive reserve in old age (Curtis et al., 2015).

### 4.2. Inflammation and cognition

No association emerged between inflammatory markers and cognitive performance in our sample. This finding is consistent with previous cross-sectional studies in healthy elderly people that investigated associations between cognitive performance and CRP (Dik et al., 2005; Wright et al., 2006; Alley et al., 2008; Trollor et al., 2012; Fard et al., 2020), IL-1 (Wright et al., 2006; Baune et al., 2008; Trollor et al., 2012; Fard et al., 2020), IL-6 (Dik et al., 2005; Alley et al., 2008; Baune et al., 2008; Fard et al., 2020) or TNF-α (Yaffe et al., 2003; Baune et al., 2008; Fard et al., 2020). However, our study contrasts with other studies that have reported positive associations in comparable samples (Weaver et al., 2002; Yaffe et al., 2003; Ravaglia et al., 2005; Wright et al., 2006; Schram et al., 2007; Trollor et al., 2012; Todd, 2017).

Several hypotheses have been advanced to explain the heterogeneous results regarding the relationship between inflammation and cognitive decline. While some authors suggest that inflammatory processes are linked with cognition in old age (Sartori et al., 2012), others argue that the role of inflammation in cognitive decline could be restricted to pathology (e.g., AD) (Todd, 2017). Indeed, most of the observed relationships between inflammation and cognition may be due to incident dementia rather than early subtle age-related cognitive decline (Alley et al., 2008).

### 4.3. Personality traits and inflammation

Likewise, no associations emerged between inflammatory markers and personality traits, which contrasts with a meta-analysis of six studies including samples of different ages (mean age ± SD: from 39.3 ± 14.7 to 72.8 ± 6.7) (Luchetti et al., 2014). The meta-analytic results revealed negative associations between Conscientiousness and both CRP and IL-6. Furthermore, the meta-analytic results indicate negative associations of Openness with CRP. Notably, these associations did not vary across age. Our results also differ from previous research by Wagner et al. (2019) which reported significant associations between Extraversion, Openness, Conscientiousness, and IL-6 levels. The discrepancies between the two studies might be explained by differences in sample size and sample characteristics (the mean age is approximately 13 years younger in their study).

Only two studies have investigated the personality-inflammation association in people over 65 years of age (Chapman et al., 2011; Mõttus et al., 2013). Chapman et al. found Conscientiousness and Openness to be negatively associated with IL-6 across three measurements taken over 34 weeks (Chapman et al., 2011), whereas Mõttus et al., using a cross-sectional design, did not find any correlation (Mõttus et al., 2013). These inconsistent results may be related to methodological aspects. Similar to our study, Mõttus et al. controlled for acute inflammation by excluding participants with a CRP of >10 m/l. One might hypothesize that the exclusion of these participants has concomitantly led to the exclusion of participants with high IL-6 levels (given that CRP production is induced by that cytokine) (Castell et al., 1990; Yap et al., 1991; Calabró et al., 2003), which reduced sensitivity. Moreover, Mõttus et al. have reported inconsistent cross-sectional results between personality and inflammation, depending on the personality assessment tool. While they found a negative association between Conscientiousness and CRP when personality was assessed using the International Personality Item Pool (IPIP) instrument, no association was observed with the NEO Five-Factor Inventory (NEO-FFI). This finding is consistent with our results since the NEO-FFI and the presently used NEO-FFI-R rely on the same model structure (McCrae and Costa, 2004).

## 5. Limitations

Several limitations should be considered when interpreting the results of the present study. First, we used a cross-sectional design that did not allow us to establish the direction of the established associations. Second, our cohort consists of an urban sample from a high-income country. Considering the high global cognitive level in such a sample (with 66.4% of participants reaching the maximal MMSE score of 30), our cognitive tests may have lacked sensitivity in detecting subtle cognitive deficits due to ceiling effects. This could explain why we observed larger cognitive associations with the verbal fluency task, a test that was not constrained by a ceiling effect. Third, blood samples were collected as part of the physical investigation, which occurred approximately 1 year before the psychiatric evaluation. Although personality traits and cognitive functioning are rather stable measures, especially during such a short period of time, it is possible that this time lag slightly diminished the size of the associations between inflammation markers and the variables assessed in the psychiatric evaluation.

## 6. Conclusion

Using a network analysis approach, we bring new insight regarding inter-relationships between cognition, personality, inflammation, and covariates. In elderly people without dementia, our findings suggest that personality is associated with cognitive abilities. In particular, individuals with a high degree of Openness exhibit better executive functioning, or semantic memory, while those with a high degree of Agreeableness are characterized by greater episodic memory abilities.

## Data availability statement

The data analyzed in this study is subject to the following licenses/restrictions: the data of CoLaus|PsyCoLaus study used in this article cannot be fully shared as they contain potentially sensitive personal information on participants. According to the Ethics Committee for Research of the Canton of Vaud, transferring or directly sharing this data would be a violation of the Swiss legislation aiming to protect the personal rights of participants. However, non-identifiable, individual-level data are available for all interested researchers, who meet the criteria for access to confidential data sharing, from the CoLaus|PsyCoLaus Datacenter (CHUV, Lausanne, Switzerland). Any researcher affiliated to an academic institution or a private research company who complies with the CoLaus|PsyCoLaus study standards for the submission of a research project can electronically submit a research application to research.colaus@chuv.ch or research.psycolaus@chuv.ch. Proposals requiring baseline data only, will be evaluated by the baseline (local) Scientific Committee SC of the CoLaus and PsyCoLaus studies. Proposals requiring follow-up data will be evaluated by the follow-up (multicentric) SC of the CoLaus|PsyCoLaus cohort study. Detailed instructions for gaining access to the CoLaus|PsyCoLaus data used in this study are available at www.colaus-psycolaus.ch/professionals/how-to-collaborate/. Requests to access these datasets should be directed to research.colaus@chuv.ch or research.psycolaus@chuv.ch.

## Ethics statement

The studies involving human participants were reviewed and approved by the institutional Ethics Committee of the University of Lausanne, which afterwards became the Ethics Commission of the Canton of Vaud (www.Cer-vd.ch) approved the baseline CoLaus|PsyColaus study (reference 16/03). The approval was renewed for the first (reference 33/09) and the second (reference 26/14) follow-ups. The study was performed in agreement with the Helsinki declaration and its former amendments, and in accordance with the applicable Swiss legislation. All participants signed a written informed consent. The patients/participants provided their written informed consent to participate in this study.

## Author contributions

TB, L-FL, IR, MT, J-MD, AvG, MP, and RR: conception and design of the study. TB, L-FL, RR, IR, and J-MD: data analysis/interpretation. TB, L-FL and RR: drafting the article. TB, L-FL, IR, MT, J-MD, M-PS, Td’A, AvG, MP, and RR: revising the article. All authors contributed to the article and approved the submitted version.

## Funding

The CoLaus|PsyCoLaus study is supported by research grants from GlaxoSmithKline, the Faculty of Biology and Medicine of the University of Lausanne, and the Swiss National Science Foundation (grants 3200B0–105993, 3200B0-118308, 33CSCO-122661, 33CS30-139468, 33CS30-148401, 33CS30_177535, and 3247730_204523) and the Swiss Personalized Health Network (project: Swiss Ageing Citizen Reference).

## Conflict of interest

The authors declare that the research was conducted in the absence of any commercial or financial relationships that could be construed as a potential conflict of interest.

## Publisher’s note

All claims expressed in this article are solely those of the authors and do not necessarily represent those of their affiliated organizations, or those of the publisher, the editors and the reviewers. Any product that may be evaluated in this article, or claim that may be made by its manufacturer, is not guaranteed or endorsed by the publisher.

## Supplementary material

The Supplementary material for this article can be found online at:

https://www.frontiersin.org/articles/10.3389/fnagi.2023.1093323/full#supplementary-material
