# Supplementary material for: Network analysis of the associations between personality traits, cognitive functioning, and inflammatory markers in elderly individuals without dementia
Source: Front Aging Neurosci. 2023 Apr 17;15:1093323. doi: 10.3389/fnagi.2023.1093323 (PMC10166137; doi:10.3389/fnagi.2023.1093323)
Supplement: Supplementary file 1 [file Data_Sheet_1.docx]

Supplementary Material

**CONTENTS**

Supplementary Table 1. Missing values and data transformation

Supplementary Table 2. Summary of bootstrap results for network A

Supplementary Table 3. Summary of bootstrap results for network B

Assessment of Inflammatory Biomarkers

Supplementary Figure 1. Network A — bootstrapped confidence intervals (CIs) of estimated edge-weights

Supplementary Figure 2. Network B — bootstrapped confidence intervals (CIs) of estimated edge-weights

Supplementary Figure 3. Spearman correlation coefficients between inflammatory markers

**Supplementary Table 1.** Missing values (MV), N = 720 participants.

| Domain | Variable | MV, n (%) |
| --- | --- | --- |
| Cognition | FCSR Free recall 1 | 31 (4.3) |
|  | FCSR Free recall 2 | 38 (5.3) |
|  | FCSR Free recall 3 | 39 (5.4) |
|  | Verbal Fluency semantic | 36 (5.0) |
|  | Verbal Fluency phonemic | 36 (5.0) |
|  | STROOP interference | 6 (0.8) |
|  | DO40 | 27 (3.8) |
|  | MMSE | None |
| Inflammation markers | IL-6 | 34 (4.7) |
|  | IL-1β | 35 (4.9) |
|  | TNF-α | 33 (4.6) |
|  | CRP | None |
| Personality | Neuroticism | None |
|  | Extraversion | None |
|  | Openness | None |
|  | Agreeableness | None |
|  | Conscientiousness | None |
| Covariates | Sex | None |
|  | Age | None |
|  | Ethnicity | None |
|  | Education | 37 (5.1) |
|  | CES-D | 70 (9.7) |
|  | Alcohol | None |
|  | Physical activity | 94 (13.1) |
|  | Smoking | 3 (0.4) |
|  | Waist circumference | 3 (0.4) |
|  | Hip circumference | 3 (0.4) |

*Note*. FCSR = Free and Cued Selective Reminding test; DO40 = Picture-naming test; MMSE = Mini-Mental State Examination; IL-6 = Interleukin-6; IL-1β = Interleukin-1β; TNF-α = Tumor Necrosis Factor-α; CRP = C-reactive protein; CES-D = Center for Epidemiologic Studies Depression scale.

**Supplementary Table 2.** Summary of bootstrapped results in network A (only non-zero edges are reported). Edges are ranked by absolute weight. “Prop0” indicates the proportion of times parameters were estimated to be zero.

|  |  |  | Bootstrap results | | | |
| --- | --- | --- | --- | --- | --- | --- |
| Edge | | sample | mean | CI lower | CI upper | Prop0 |
| Verbal Fluency | DO40 | 0.31 | 0.31 | 0.16 | 0.47 | 0.4 |
| Free Recall | Verbal Fluency | 0.30 | 0.30 | 0.23 | 0.37 | 0 |
| IL-6 | TNF-α | 0.26 | 0.27 | 0.19 | 0.33 | 0 |
| Extraversion | Conscientiousness | 0.24 | 0.24 | 0.16 | 0.31 | 0 |
| Neuroticism | Extraversion | -0.24 | -0.25 | -0.32 | -0.17 | 0 |
| IL-6 | IL-1β | 0.22 | 0.23 | 0.14 | 0.30 | 0 |
| Neuroticism | Conscientiousness | -0.21 | -0.21 | -0.28 | -0.13 | 0 |
| Free Recall | MMSE | 0.21 | 0.18 | 0.08 | 0.34 | 1.4 |
| Agreeableness | Conscientiousness | 0.20 | 0.21 | 0.12 | 0.28 | 0 |
| Verbal Fluency | Openness | 0.18 | 0.18 | 0.11 | 0.24 | 0 |
| Extraversion | Openness | 0.17 | 0.16 | 0.09 | 0.24 | 0.1 |
| Stroop | DO40 | 0.14 | 0.09 | -0.09 | 0.37 | 55.3 |
| IL-1β | TNF-α | 0.13 | 0.13 | 0.05 | 0.21 | 0.7 |
| Stroop | Openness | 0.09 | 0.06 | -0.04 | 0.22 | 44.9 |
| Free Recall | Agreeableness | 0.09 | 0.09 | 0.01 | 0.17 | 5.8 |
| Verbal Fluency | MMSE | 0.08 | 0.08 | -0.05 | 0.20 | 24.4 |
| Neuroticism | Agreeableness | -0.08 | -0.08 | -0.16 | 0.01 | 9.6 |
| Verbal Fluency | Stroop | 0.07 | 0.06 | -0.05 | 0.18 | 39.3 |
|  |  |  |  |  |  |  |

*Note*. DO40 = Picture-naming test; MMSE = Mini-Mental State Examination; IL-6 = Interleukin-6; IL-1β = Interleukin-1β; TNF-α = Tumor Necrosis Factor-α; CRP = C-reactive protein.

**Supplementary Table 3.** Summary of bootstrapped results in network B (only non-zero edges are reported). Edges are ranked by weight. “Prop0” indicates the proportion of times parameters were estimated to be zero.

|  |  |  | Bootstrap results | | | |
| --- | --- | --- | --- | --- | --- | --- |
| Edge | | sample | mean | CI lower | CI upper | Prop0 |
| Male sex | WHR | 0.85 | 0.89 | 0.67 | 0.99 | 0 |
| Male sex | Education | 0.55 | 0.52 | 0.32 | 0.78 | 0.1 |
| Male sex | Alcohol | 0.43 | 0.44 | 0.31 | 0.56 | 0 |
| Neuroticism | CES-D | 0.43 | 0.43 | 0.37 | 0.49 | 0 |
| Free Recall | Male sex | -0.37 | -0.35 | -0.52 | -0.23 | 0 |
| Agreeableness | Male sex | -0.35 | -0.35 | -0.50 | -0.21 | 0 |
| Verbal Fluency | DO40 | 0.30 | 0.28 | 0.15 | 0.46 | 1.0 |
| Free Recall | Verbal Fluency | 0.29 | 0.28 | 0.22 | 0.35 | 0 |
| IL-6 | TNF-α | 0.25 | 0.26 | 0.18 | 0.32 | 0 |
| MMSE | Male sex | -0.23 | -0.20 | -0.48 | 0.02 | 20.6 |
| Extraversion | Conscientiousness | 0.23 | 0.23 | 0.16 | 0.30 | 0 |
| IL-6 | IL-1β | 0.21 | 0.21 | 0.13 | 0.28 | 0 |
| Agreeableness | Conscientiousness | 0.20 | 0.19 | 0.12 | 0.27 | 0 |
| Neuroticism | Extraversion | -0.19 | -0.19 | -0.26 | -0.12 | 0 |
| Free Recall | Education | 0.17 | 0.12 | 0.04 | 0.31 | 12.9 |
| Neuroticism | Conscientiousness | -0.17 | -0.16 | -0.24 | -0.09 | 0.1 |
| Free Recall | MMSE | 0.17 | 0.14 | 0.04 | 0.29 | 5.8 |
| Verbal Fluency | Openness | 0.17 | 0.16 | 0.10 | 0.23 | 0 |
| Extraversion | Openness | 0.16 | 0.15 | 0.09 | 0.23 | 0.1 |
| Openness | Education | 0.14 | 0.12 | 0.01 | 0.28 | 11.8 |
| Neuroticism | Male sex | -0.13 | -0.11 | -0.32 | 0.06 | 37.8 |
| Verbal Fluency | Education | 0.13 | 0.11 | 0.00 | 0.25 | 13.5 |
| IL-1β | TNF-α | 0.11 | 0.12 | 0.03 | 0.19 | 1.9 |
| Verbal Fluency | MMSE | 0.10 | 0.08 | -0.02 | 0.22 | 29.4 |
| Neuroticism | Agreeableness | -0.10 | -0.08 | -0.21 | 0.01 | 26.7 |
| Free Recall | AGE | -0.10 | -0.10 | -0.18 | -0.02 | 6.6 |
| CRP | WHR | 0.09 | 0.12 | -0.01 | 0.20 | 9.4 |
| Free Recall | Agreeableness | 0.05 | 0.03 | -0.02 | 0.13 | 51.9 |

*Note*. DO40 = Picture-naming test; MMSE = Mini-Mental State Examination; IL-6 = Interleukin-6; IL-1β = Interleukin-1β; TNF-α = Tumor Necrosis Factor-α; CRP = C-reactive protein; CES-D = Center for Epidemiologic Studies Depression scale; WHR = Waist-to-hip ratio.

**Assessment of Inflammatory Biomarkers**

Morning venous blood samples (50 mL) were drawn in the fasting state and allowed to clot. Serum was preferred to plasma, as it has been shown that different anticoagulants may differentially affect absolute cytokine levels (Flower et al., 2000; Skeppholm et al., 2008). High-sensitivity C-reactive protein (CRP) was assessed by immunoassay and latex HS (IMMULITE 1000- High, Diagnostic Products Corporation, LA, CA, USA) with maximum intra- and inter-batch coefficients of variation (CV) of 1.3% and 4.6%, respectively. Serum samples were kept at –80°C before assessment of interleukin (IL)-1β, IL-6, and Tumor Necrosis Factor -α (TNF-α), sent on dry ice to the laboratory, and only subjected to a single freeze/thaw cycle before analysis. Levels of these cytokines were measured using a multiplexed particle-based flow cytometric cytokine assay (Vignali, 2000). This methodology yields cytokine concentrations correlating well with those obtained by other methods such as ELISA (dupont et al., 2005; Elshal and McCoy, 2006). Milliplex kits were purchased from Millipore (Zug, Switzerland). The procedures closely followed the manufacturer’s instructions. The analysis was conducted using a conventional flow cytometer (FC500 MPL, BeckmanCoulter, Nyon, Switzerland). Good agreement between signal and cytokine was found within the assay range (R^2^ ≥ 0.99). Intra- and inter-assay CV were respectively 15% and 16.7% for IL-1β, 16.9% and 16.1% for IL-6 and 12.5% and 13.5% for TNF-α. For quality control, repeated measurements were conducted in 80 subjects randomly drawn from the initial sample. “Spearman rank correlations (n=80) between duplicate measurements were 0.914, 0.961, and 0.891 for IL-1β, IL-6, and TNF-α (all p < 0.001), respectively, while Lin’s correlation coefficients were 0.969, 0.971, and 0.945 and intra-class correlation coefficients were 0.970, 0.972, and 0.946 for IL-1β, IL-6, and TNF-α, respectively (all p < 0.001), indicating a good reproducibility.” (Marques-Vidal et al., 2011).

Lower limits of detection (LOD) for IL-1β, IL-6 and TNF-α were 0.2 pg/ml. Undetectable measures for IL-1β, IL-6 and TNF-α were replaced by half the LOD (i.e., 0.1 pg/ml), as was previously suggested (Vignali, 2000; dupont et al., 2005; Elshal and McCoy, 2006). For IL-6 and TNF-α, all values were detectable. IL-6 showed a median of 2.56 pg/ml (interquartile range (IQR): 1.06–7.76), IL-1β of 0.43 pg/ ml (IQR: 0.10–1.73), TNF-α of 4.90 pg/ml (IQR: 2.96–8.28), and CRP of 1.60 mg/l (IQR: 0.80–3.00). Spearman’s correlation coefficients between the different inflammatory markers are reported in Supplementary Table 1.


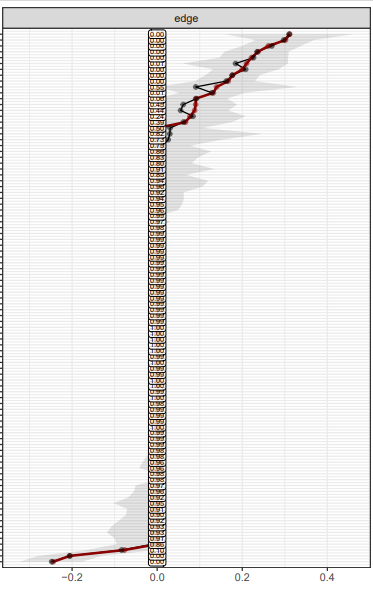


**Supplementary Figure 1.** Network A — Bootstrapped confidence intervals (CIs) of estimated edge-weights. The red line indicates the sample values and the grey area the bootstrapped CIs. Each horizontal line represents one edge of the network, ordered from the edge with the highest weight to the edge with the lowest weight. In the case of ties (for instance, multiple edge-weights were estimated to be exactly 0), the mean of the bootstrap samples was used in ordering the edges. The y-axis labels have been removed to avoid cluttering.


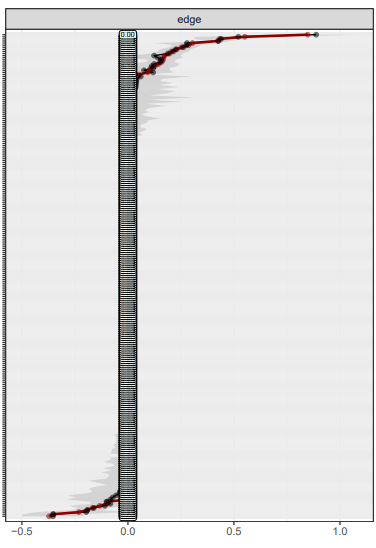


**Supplementary Figure 2.** Network B — Bootstrapped confidence intervals (CIs) of estimated edge-weights. The red line indicates the sample values and the grey area the bootstrapped CIs. Each horizontal line represents one edge of the network, ordered from the edge with the highest weight to the edge with the lowest weight. In the case of ties (for instance, multiple edge-weights were estimated to be exactly 0), the mean of the bootstrap samples was used in ordering the edges. The y-axis labels have been removed to avoid cluttering.

**
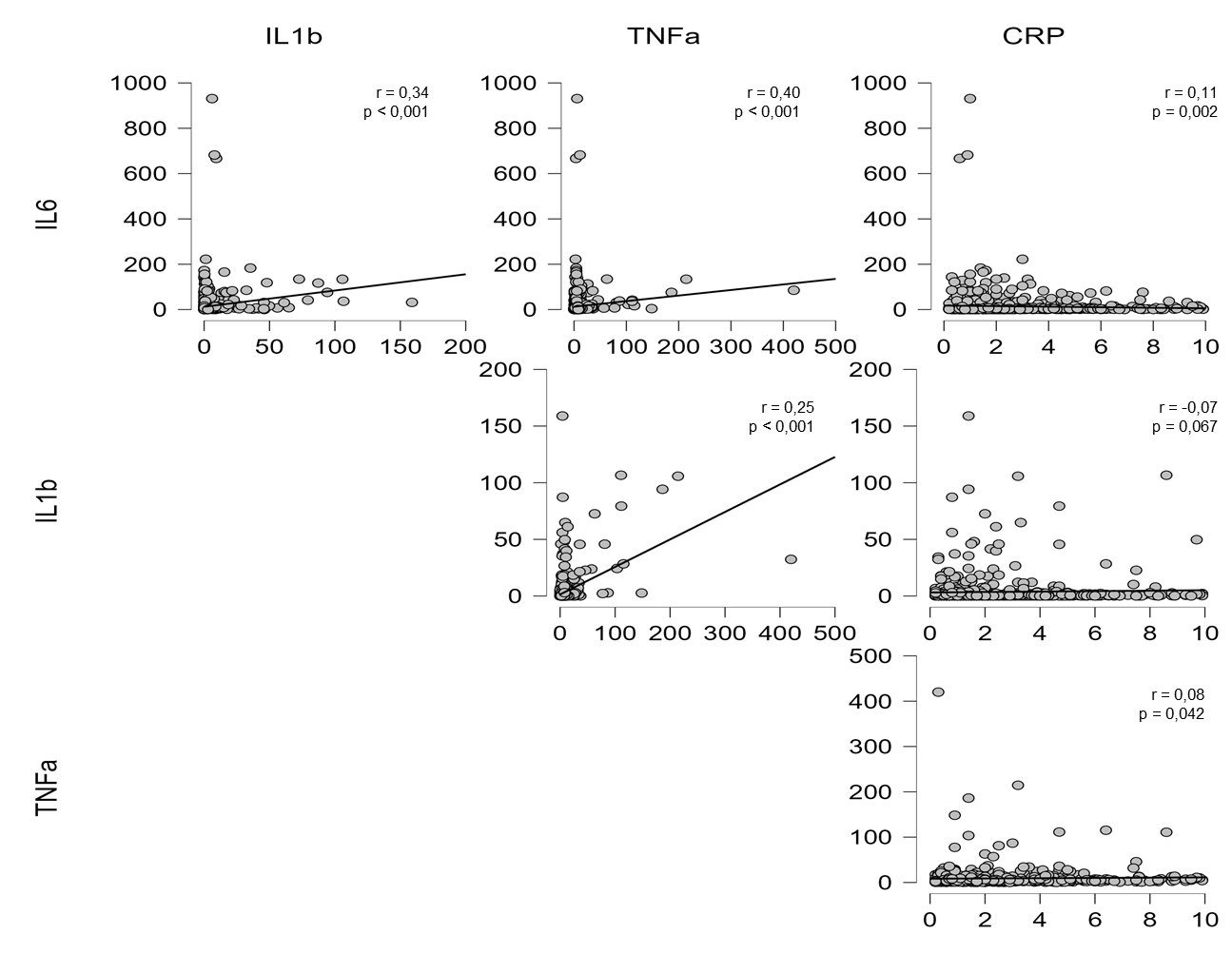
**

**Supplementary Figure 3.** Spearman correlation coefficients between inflammatory markers (computed on raw values). IL, interleukin (pg/ml); TNF, tumor necrosis factor (pg/ml); CRP, C-reactive protein (mg/ml).

**References**

dupont, N. C., Wang, K., Wadhwa, P. D., Culhane, J. F., and Nelson, E. L. (2005). Validation and comparison of luminex multiplex cytokine analysis kits with ELISA: determinations of a panel of nine cytokines in clinical sample culture supernatants. *J. Reprod. Immunol.* 66, 175–191. doi: 10.1016/j.jri.2005.03.005.

Elshal, M. F., and McCoy, J. P. (2006). Multiplex bead array assays: performance evaluation and comparison of sensitivity to ELISA. *Methods San Diego Calif* 38, 317–323. doi: 10.1016/j.ymeth.2005.11.010.

Flower, L., Ahuja, R. H., Humphries, S. E., and Mohamed-Ali, V. (2000). EFFECTS OF SAMPLE HANDLING ON THE STABILITY OF INTERLEUKIN 6, TUMOUR NECROSIS FACTOR-α AND LEPTIN. *Cytokine* 12, 1712–1716. doi: 10.1006/cyto.2000.0764.

Marques-Vidal, P., Bochud, M., Bastardot, F., Lüscher, T., Ferrero, F., Gaspoz, J.-M., et al. (2011). Levels and determinants of inflammatory biomarkers in a Swiss population-based sample (CoLaus study). *PloS One* 6, e21002. doi: 10.1371/journal.pone.0021002.

Skeppholm, M., Wallén, N. H., Blombäck, M., and Kallner, A. (2008). Can both EDTA and citrate plasma samples be used in measurements of fibrinogen and C-reactive protein concentrations? *Clin. Chem. Lab. Med.* 46, 1175–1179. doi: 10.1515/CCLM.2008.219.

Vignali, D. A. (2000). Multiplexed particle-based flow cytometric assays. *J. Immunol. Methods* 243, 243–255. doi: 10.1016/s0022-1759(00)00238-6.
